# Benchmark findings from a veteran electronic patient-reported outcomes evaluation from a chronic pain management telehealth program

**DOI:** 10.1186/s12913-024-10816-4

**Published:** 2024-03-28

**Authors:** Jolie N. Haun, Christopher A. Fowler, Bridget M. Smith, Lishan Cao, Kevin T. Stroupe, William A. Lapcevic, Michael S. Saenger, Rachel C. Benzinger, Dustin D. French

**Affiliations:** 1 Research and Development Service, James A. Haley Veterans’ Hospital, 8900 Grand Oak Circle, Tampa, FL 33637 USA; 2 Division of Epidemiology, Department of Internal Medicine, University of Utah, 295 Chipeta Way, Salt Lake City, UT 84132 USA; 3 Department of Psychiatry and Behavioral Neurosciences, University of South Florida, 3515 E. Fletcher Ave., Tampa, FL 33613 USA; 4 Center of Innovation for Complex Chronic Healthcare, Department of Veterans Affairs, Edward Hines, Jr. VA Hospital, 5000 South 5th Ave., Hines, IL 60141 USA; 5 Anesthesia Service Line, Atlanta Veterans Administration Health Care System, 1670 Clairmont Rd., Decatur, GA 30033 USA; 6 Division of Internal Medicine, School of Medicine, Emory University, 201 Dowman Dr., Atlanta, GA 30322 USA; 7 Center for Health Services and Outcomes Research, Feinberg School of Medicine, Northwestern University, 633 N. St. Clair, St. Suite 2000, Chicago, IL 60611 USA; 8 Departments of Ophthalmology and Medical Social Sciences, Feinberg School of Medicine, Northwestern University, 645 N. Michigan Ave., Suite 440, Chicago, IL 60611 USA

**Keywords:** Chronic pain, Pain management, Opioids, Benzodiazepines, Veterans, Acceptance and Commitment Therapy (ACT), Mindful movement, Whole health

## Abstract

**Background:**

Chronic pain is a leading cause of disability and negatively impacts biological/physical, psychological, and social aspects of life resulting in significant pain interference or disability. This project was part of a longitudinal mixed-methods implementation evaluation of the TelePain-Empower Veterans Program (EVP), a non-pharmacological chronic pain intervention. The purpose of this quality management project was to examine electronic patient-reported outcome measures (ePROs) including primary pain-related (intensity, interference, catastrophizing, kinesiophobia) and secondary outcomes (physical, psychological, acceptance, social) to determine TelePain-EVP effectiveness. Secondary purpose was to examine dosing effects to better understand potential dose relationships between EVP use and ePROs.

**Methods:**

Standardized ePRO measures were examined at week 1 (baseline), week 10 (post-EVP), and week 26 (follow-up). Qualtrics, a cloud-based platform was used to collect ePRO data at each time point. Veterans that completed at-least one survey at any specified time point were categorized as responders (*n* = 221). Linear-mixed models (LMMs) were fit to assess changes for each primary and secondary ePRO.

**Results:**

Participants ranged from 24 to 81 years old; veterans were typically male (65.16%), black or African American (76.47%), married or partnered (41.63%), attended at-least some college or vocational school (67.87%), and reported low back as their primary pain location (29.41%). There was a significant decrease in pain catastrophizing from baseline to post-TelePain-EVP (*p* < .001). However, pain catastrophizing improvement from baseline was not present at week 26 (*p* = .116). Pain interference also decreased from baseline to post-treatment (*p* = .05), but this improvement did not exceed the adjusted significance threshold. Additional pre-post improvements were also observed for certain secondary ePROs: psychological (anxiety, depression), acceptance (activities engagement). Only the activities engagement effect remained 26 weeks from baseline. Mixed results were observed for EVP dose across primary and secondary outcomes.

**Conclusions:**

Evidence from this evaluation indicate that TelePain-EVP has positive outcomes for certain pain (catastrophizing), psychological (anxiety, depression), and acceptance (activities engagement) for veterans with chronic pain. More TelePain related studies and enterprise-wide evaluations are needed along with comparative and cost effectiveness methods to determine patient benefits and the economic value gained of treatment options such as TelePain-EVP.

**Supplementary Information:**

The online version contains supplementary material available at 10.1186/s12913-024-10816-4.

## Background

Chronic pain is a leading cause of disability [1], impacting more the 50 million American adults [2]. As defined, chronic pain persists beyond 3-to-6 months following its initial onset [3]. Chronic pain can negatively impact the biological/physical, psychological, and social areas of an individual’s life resulting in significant pain interference or disability in their functioning and daily lives (high-impact chronic pain) [4, 5]. Estimates suggest that a quarter to a third or more of chronic pain cases may be classified as high impact [2, 6].

There are higher rates of overall (29.1% v. 19.5%) and high-impact (9.1% v. 6.4%) chronic pain in the veteran population versus the general public [2, 7]. Incidence of chronic pain may be even higher among combat veterans with estimates of up to 81.5% among Operation Enduring Freedom and Iraqi Freedom veterans [8]. The high prevalence of chronic pain in the veteran population can also contribute to the psychological health (e.g., depression, anxiety, sleep, posttraumatic stress disorder) and substance use disorders burden given that these are common comorbidities among people with chronic pain [9–12]. Furthermore, there may also be a dose–response relationship between increased veteran pain severity and completed suicide after accounting for demographic and psychological factors [13]. Given the significant burden and risk factor that chronic pain poses for veterans, it has been designated as a high priority area by Department of Veterans Affairs (VA) [14, 15].

Over reliance on the use of prescription opioids as a frontline treatment for chronic pain contributed to a national epidemic including increased rates of opioid addiction, accidental overdose, and even mortality [16–20]. Dangerous drug interactions, notably concurrent opioid and benzodiazepine use, have further exacerbated these issues. An analysis of the Drug Abuse Warning Network and the National Vital Statistics System databases from 2004–2011 revealed that concurrent opioid and benzodiazepine use was associated with significant increases in both emergency department visits (from 11.0 to 34.2 per 100,000) and overdose deaths (from 0.6 to 1.7 per 100,000) [21]. A random sample of over 420,000 veterans that were prescribed opioids from 2004–2009 revealed that 26.7% had also been prescribed benzodiazepines. Of the approximately 2,400 who died from a drug overdose, roughly half (*n*= 1,185) were also prescribed benzodiazepines [22]. Such alarming findings and changes in guidelines for prescription opioid use have led to decreases in prescribing trends nationally [17, 23].

The 2016 Comprehensive Addiction and Recovery ACT mandated that the VA limit the use of long-term opioid prescribing for chronic pain management [24, 25]. Current (2022) VA/Department of Defense (DoD) guidelines recommend: 1) the use of non-pharmacological treatments for veterans not currently prescribed opioids; 2) biopsychosocial assessment to determine whether benefits outweigh risks before starting veterans on prescription opioids; 3) biopsychosocial assessment to determine the appropriateness of opioid taper, discontinuation, or prescription change for veterans currently prescribed opioids; and 4) that interdisciplinary pain care teams consisting of integrated providers (e.g., psychology, physical therapy, nursing, etc.) are an ideal treatment option when available [26]. The VA/DoD guidelines continue to recommend investigation of interdisciplinary chronic pain care as a research priority.

Consistent with the VA’s mission to address chronic pain as a biopsychosocial condition, contemporary treatment approaches including Acceptance and Commitment Therapy (ACT) expand their focus beyond reducing pain severity alone [14, 27, 28]. Instead, ACT focuses on helping the individual lead a fulfilling life despite the presence of chronic pain-related discomfort. Re-engaging in values-driven actions including important life roles (e.g., family, occupational, social) can facilitate opportunities to improve their overall quality of life and functioning. Hence, ACT may indirectly reduce pain interference [29, 30]. ACT has shown effectiveness for functional and quality of life improvements when interventions directly targeting nociceptive pain achieve limited success [30, 31].

The effectiveness of integrated interdisciplinary chronic pain programs on improving multi-dimensional pain outcomes is well-established in VA settings [32–36]. The Empower Veterans Program (EVP) is a non-pharmacological interdisciplinary chronic pain rehabilitation program that integrates ACT, its core behavioral therapy, with mindful movement (MM), and whole health (WH) [36]. MM within EVP teaches veterans to observe and accept mind–body experiences while emphasizing an open, nonjudgmental attitude [37]. The focus is to change their relationship between unpleasant thoughts, emotions, and body sensations than can influence pain (e.g., willing engagement in previously avoided movements due to reduced fear or sense of threat). WH integrates complimentary and integrative modalities into conventional healthcare approaches to offer a patient-centered, values-driven approach to veteran health [38]. Within EVP, WH allows veterans to develop personalized health plans that focus on physical, psychological, environmental, and spiritual health-related factors (e.g., sleep, nutrition, relationships) that may influence pain outcomes [39].

An early EVP qualitative evaluation found that veterans described adopting new self-care and lifestyle practices for pain management, pain acceptance, life participation, changing medication use, and greater ability to adjust to life’s challenges [39]. A pre-post pilot evaluation [40] found medium-to-large effect size improvements in pain intensity, catastrophizing, and health-related quality of life (HRQoL) among veterans that graduated (attended ≥ 8 of 10 sessions) from EVP. Veterans also reported high program satisfaction [40]. A larger pre-post quality management project [36] found clinical small-to-medium effect size improvements in primary outcomes including pain intensity, interference, and catastrophizing. Small-to-medium effect size improvements were also observed for 12 of 17 secondary outcomes including physical (fatigue), psychological (anxiety, depression, sleep disturbance), and HRQoL (environmental, physical, psychological, social) domains. Small-to-large effect size improvements for ACT (activities engagement, pain willingness) and mindfulness (nonreactivity to thoughts/emotions, sensory observation) domains also provided support for EVP’s theoretical foundations in ACT and MM. Interestingly, pre-post clinical improvements did not differ between veterans that graduated from EVP compared to non-graduates. Veterans also provided high favorability ratings for EVP [36]. Findings from these evaluations suggest that EVP is a workable non-pharmacological interdisciplinary rehabilitation option for veterans with chronic pain [36, 39, 40].

Positive support for EVP notwithstanding, several important questions remain to be addressed in the current study. First, EVP evolved into a telehealth program (TelePain-EVP) in response to the COVID-19 pandemic which aligned VA’s mission to expand veteran access to telehealth services [36, 41, 42]. To date, EVP has been evaluated as an in-person program and how its benefits may translate to a telehealth platform warrant investigation. Second, in the previous evaluations, EVP graduation was operationalized as a binary variable [36, 40]. However, in the largest EVP evaluation to date, graduation status did not predict pre-post improvements for any primary or secondary outcomes when adjusting for family-wise error rate [36]. To examine potential dosing effects for EVP, examining dose on a continuum beyond a simple binary measure is warranted. Third, despite positive effects associated with EVP participation, certain contradictory findings were observed. Specifically, while improvements were observed for physical and social HRQoL, the related domains of physical functioning and social roles actually decreased from pre-post EVP [36]. Finally, previous EVP evaluations have examined pre-post changes in veteran outcomes, but no studies have examined follow-up time points to examine robustness of clinical improvements. Further examination may help with interpreting the robustness of these and other findings.

The current longitudinal evaluation, part of a larger implementation evaluation effort, followed a veteran cohort who initiated in TelePain-EVP at a large VA Medical Center in the southeast. The purpose of this quality management project are as follows. First, to examine electronic patient-reported outcome measures (ePROs) including primary pain-related (pain intensity, interference, catastrophizing, kinesiophobia) and secondary outcomes (physical, psychological, ACT, social) to determine TelePain-EVP effectiveness. Second, TelePain-EVP dosing effects were examined to better understand any potential dose relationships between EVP use and ePROs. Third, to describe post-TelePain-EVP satisfaction. This evaluation of TelePain-EVP can support the VA’s mission to provide innovative non-pharmacological and telehealth programs for interdisciplinary chronic pain management.

## Methods

### Design

As part of a larger quality improvement implementation and evaluation effort, this project leveraged a within-participants repeated-measured design to examine veteran ePROs across four time points [weeks: 1 (baseline), 10 (post-EVP), 26 (follow-up 1), 52 (follow-up 2)].

### EVP intervention

TelePain-EVP is an evidence-based program delivered in a weekly format over 10 consecutive weeks [36, 37, 39, 40, 43]. Each week, veteran cohorts (*n* = 4 to 20) engage in three one-hour evidence-informed group therapy sessions facilitated by an integrated interdisciplinary team of professionals (ACT – psychologists/social workers, MM – physical therapists, WH – chaplains). In total, the program offers 30 hourly sessions in addition to weekly in-home practices to be used in daily life. Participants also receive weekly motivational interviewing-driven coaching calls with the WH chaplains. TelePain-EVP uses a standardized format to optimize session fidelity. For a more comprehensive review of EVP, see Haun et al. [36] A breakdown of TelePain-EVP sessions by therapy type is shown in Table 1.

**Table 1:** Sample 10-week curriculum for the TelePain-Empower Veterans Program (EVP)

Week	EVP Core Components and Weekly Objectives		
	**EVP ACT**	**EVP MM**	**EVP WH**
1	• Introductions • Reviewing group guidelines • Overview of ACT	• Introductions • Pain & the Brain Part 1	• Introductions • Overview of WH
2	• Exploring personal values and life purpose	• Pain & the Brain Part 2 • Neutral Spine	• Introducing Mindful Awareness • Power of the Mind
3	• Metaphors exploring Psychological Flexibility	• Motion is Lotion Exercises (MILES) 1 • Hand motion	• Mindfulness practices • Food and Drink
4	• Noticing added suffering from current avoidance/ coping strategies	• MILES 1 & 2 • Head and eyes motion	• Mindfulness practices • Recharge/Sleep
5	• Defusion; Tricks of the mind	• MILES 1—3 • Feet and foot motion	• Observer Self practice • Choice of gratitude
6	• Cycle: Behaviors, Thoughts, and Emotions	• MILES 1—4 • Core motion • Computer workstation	• Self-Compassion practice • Choice of Kindness
7	• Committed Action	• MILES 1, 2, 3, 4 & 5 • EVP Tai Chi (Part 1)^a^	• Self-Compassion practice • Choice of active listening in relationship building
8	• Acceptance/Willingness	• MILES 1–5 • EVP Yoga^a^	• Self-Compassion practice • Considering choice of forgiveness
9	• Maintaining progress	• MILES 1, 2, 3, 4 & 5 • EVP Tai Chi (Part 2)^a^	• Self-Compassion practice • Finding meaning in suffering
10	• Value declaration • Graduation	• EVP “Tai Chi” • Graduation	• Whole Body Scan • Graduation

*ACT* Acceptance and Commitment Therapy, *EVP* Empower Veterans Program, *MM* Mindful Movement, *WH* Whole Health

^a^Tai Chi and Yoga not provided by certified instructors but consistent with the MM portion of EVP’s programmatic modalities

### Recruitment and sample size

**Fig. 1:**
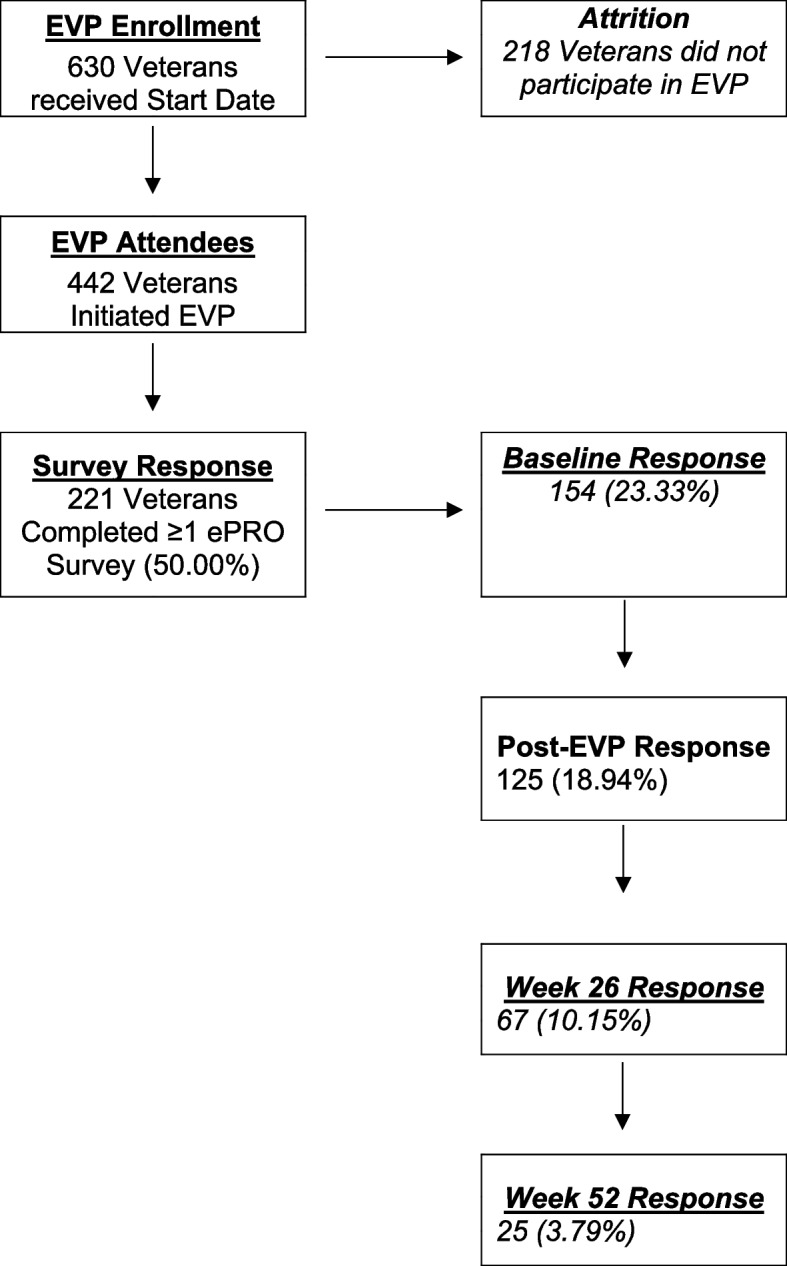
TelePain-Empower Veterans Program (EVP) admission survey response rate from 2021–2023

A cohort of 630 veterans with chronic pain that agreed to participate in TelePain-EVP were assigned a start date between December 1, 2021, and June 30, 2023, and targeted for this quality management project. Two-hundred and eighteen veterans (33.03%) did not attend TelePain-EVP and were excluded. In total, 442 of these veterans (70.16%) initiated TelePain-EVP by attending at-least one session hour of ACT, MM, or WH. Of the veterans that initiated in this TelePain-EVP, 221 (survey response rate = 50.00%) completed at-least one ePRO survey at any time point and were used as participants and were included for analysis. A flow diagram of the sampling process and survey response rate is presented in Fig. 1.

Internet access and a smart device (e.g., computer, smartphone, tablet) were required to participate in TelePain-EVP. To ensure veterans could access TelePain-EVP, a provider referral was scheduled for a pre-intervention introduction session using the Veteran Video Connect (VVC), a VA telehealth platform. Veterans that had difficulty connecting to VVC were provided with the phone number for the VA Office of Connected Care help desk for support. For veterans who did not have access to a device (e.g., homelessness), a social work consult was placed to assist the veteran with getting access to a tablet and VVC access training. Veterans' caregivers were encouraged to work with the veteran to assist with TelePain-EVP access and participation. While no formal literacy assessment was conducted, for veterans with reading comprehension difficulties, TelePain-EVP providers assisted with these challenges during weekly 1:1 coaching sessions. There were no formal TelePain-EVP exclusion criteria.

### Measures

#### Electronic patient-reported outcomes

Primary pain-related outcomes (intensity, interference, catastrophizing, kinesiophobia), as well as secondary outcomes including physical functioning, psychological health (depression, anxiety, sleep disturbance), social isolation, HRQoL, acceptance (activities engagement, pain willingness), and readiness for change (motivation, self-efficacy) were examined using validated ePRO survey measures. When available, survey short forms were used to reduce participants' response burden. Satisfaction with TelePain-EVP and global impressions of change following treatment were also examined. Table 2 presents ePRO surveys and dose measures administered to assess the TelePain-EVP.

**Table 2:** Electronic patient-reported outcome and dose measures administered to assess the TelePain-Empower Veterans Program (EVP)

Scale	Construct	Description	Items	Scale(s)
**Pain**				
Graded Chronic Pain Scale-Revised [44]	Pain Intensity	Pain intensity, interference with life enjoyment and general activity in the past week, and occupational functioning.	6	0 – *never,* 3 – *everyday*; 0 – 10^a^; 0 – *no,* 1 – *yes*
PROMIS – Pain Interference Scale [45]	Pain Interference	The impact of pain on daily functioning.	4	1 – *not at all*, 5 – *very much*
Pain Catastrophizing Scale – 3-item [46]	Pain Catastrophizing	Maladaptive and exaggerated beliefs “toward actual or anticipated” pain experiences” (p. 602) [47].	3	0 – *not at all*, 4 – *all the time*
Tampa Scale of Kinesiophobia – 4-item [48]	Kinesiophobia	Fear of movement secondary to pain.		
**Physical**				
PROMIS 4a – Physical Functioning [45]	Physical Functioning	Perceived physical capability to engage in daily activities (e.g., self-care, endurance).	4	1 – *unable to do*, 5 – without any difficulty^*^
PROMIS 4a – Sleep [45]	Sleep Disturbance	Difficulty falling and staying asleep.	4	1 – *not at all*, 5 – *very much*
**Psychological**				
Patient Health Questionnaire-4 – Anxiety subscale [45]	Anxiety	Anxiety symptom severity over the previous 2 weeks.	2	0 – *not at all*, 3 – *nearly every day*
Patient Health Questionnaire-3 – Depression Subscale [49]	Depression	Depression symptom severity over the previous 2 weeks.	9	0 – *not at all*, 3 – *nearly every day*
**Social Isolation**				
PROMIS 4a – Social Isolation [50]	Social Isolation	Perceived avoidance, exclusion, disconnectedness, or detachment from others.	4	1 – *always*, 5 – *never*
**Acceptance**				
Chronic Pain Acceptance Questionnaire-8 [51]	Activity Engagement	Engagement in life activities despite experiencing pain.	8	0 – *never true*, 6 – *always true*
	Pain Willingness	Willingness to experiences pain without attempts to control it.		
**Motivation**				
Readiness Ruler [52]	Motivation	Readiness to effectively manage chronic pain.		0 – *not at all*,
	Self-Efficacy	Confidence to manage chronic pain.		10 – *extremely*
**Program Satisfaction**				
National Veterans Health Administration – Satisfaction Survey Item [53]	EVP Satisfaction	Overall satisfaction with EVP for pain care.	1	1 – *poor*, 5 – *excellent*
**Covariates**				
Pain Outcomes Questionnaire-Veterans Affairs [54]	Demographics	Age	14	Continuous
		Gender		Nominal
		Race		White v. Non-White
		Service-Connected Disability		Yes, No
		Marital		Nominal
		Employment		Nominal
		Education		Nominal
		Rurality		Rural, Urban
		Pain Location(s)		Continuous
		Primary Location		Nominal
		Claims In Progress		Yes, No
		Service Branch		Nominal
		Service Grade		Nominal
		Combat Exposure		Yes, No

*PROMIS* Patient Reported Outcome Measurement Information System, *HRQoL* Health-Related Quality of Life

^a^Item-level response options vary by domain

#### TelePain-EVP dose

TelePain-EVP dosage units were quantified using total hours attended. Specifically, each weekly session (ACT, MM, WH) was disaggregated into three separate treatment hours one could attend. Thus, TelePain-EVP dose had a possible range from 0 to 30 hours. This differs from previous EVP evaluations which examined graduation status and examined attendance on a weekly basis versus hourly [36, 40].

### Data collection procedures

Data was collected at a large VA medical center in the southeastern United States. The Emory University Institutional Review Board reviewed the current protocol and deemed it to be a non-research quality improvement project. Prior to participation, informed consent was obtained from the participants or legally authorized representatives prior to participating in this evaluation. Data was collected using multiple methods and platforms. Data from each respective source was extracted and stored on a secure drive behind the VA firewall.

### Participant recruitment and program dose

TelePain-EVP dosing was collected using a secure customized platform. A Microsoft Access (Version 2203) [55] front end was developed to allow TelePain-EVP clinicians and administrators to enter contact information for veteran participants and attendance to each individual session (ACT, MM, WH) on a weekly basis. This database was connected to a Microsoft SQL (Server 2016) [56] back end for data management by the study team. This platform was also used to generate a unique ID number for each veteran used to de-identify the database during data extraction.

### Electronic data collection

Standardized ePRO measures were administered at the beginning of week 1 (baseline), week 10 (post-EVP), week 26 (6-months post baseline), and week 52 (12-months post baseline). Expected survey completion times were 20 min for baseline (ePROs and demographics) and 15 min for each additional time point (ePROs only). Qualtrics, a Federal Risk and Authorization Management Program-approved cloud-based data collection platform was used to collect ePRO data at each time point. Veteran e-mail addresses were extracted from the Microsoft Access/SQL platform and entered into Qualtrics for dissemination of web links to ePRO surveys (see Table 2).

Qualtrics has demonstrated usability for ePRO data collection within the VA system [57].

First, Qualtrics was used to collect ePROs which included primary pain-related (pain intensity, interference, catastrophizing, kinesiophobia) and secondary outcomes (physical, psychological, HRQoL, ACT, social) used to determine examine TelePain-EVP effectiveness. Second, Qualtrics was used to examine TelePain-EVP dosing effects to better understand any potential dose–response relationships between EVP use and ePROs. Third, Qualtrics was used to describe post-TelePain-EVP satisfaction. This evaluation of TelePain-EVP can support the VA’s mission to provide innovative non-pharmacological and telehealth programs for interdisciplinary chronic pain management.

### Statistical analysis

#### Preliminary analyses

Normally distributed continuous data were described using means and standard deviations. Skewed continuous variables were described using the medians (*mdn*) and interquartile ranges (*IQR*). Frequencies and percentages were used to describe categorical variables. Participants that completed at-least one ePRO survey at any specified time point were categorized as responders. Participant demographic characteristics are presented as covariates in Table 2.

#### Primary and secondary ePROs analyses

Linear-mixed models (LMMs) were fit to assess changes for each primary and secondary ePRO. Mixed models can handle unbalanced data by making use of available information when outcome data is available at one time point and missing at others, thus preserving sample size [58, 59]. Each LMM used an auto-regressive covariance matrix. Random intercepts were fit to account for within-participants correlations. Each model included time, dose, and their interaction as fixed effects. Non-significant interactions were dropped from models to preserve sample size and control against multicollinearity potentially inflating standard errors. To account for multiple tests, a conservative *p*-value (.01) was used to determine statistical significance for analyses of all primary and secondary outcomes. Mixed model analyses were conducted using Analyses used Proc Mixed in SAS® version 9.4 Cary, NC. Standardized mean differences (SMD) effects sizes for correlated samples were calculated to determine changes across time points using a Microsoft Excel macro [60]. Effect sizes SMDs were adopted from meta-analysis of psychological and chronic pain interventions that align with ACT and MM principles: ≤ 0.32 = small; 0.33–0.55 = moderate; ≥ 0.56 = large [61].

#### Post -TelePain-EVP satisfaction

In previous evaluations, EVP satisfaction was negatively skewed indicating higher satisfaction scores indicating the appropriateness of non-parametric tests [36, 40]. To evaluate program satisfaction, a one sample Wilcoxon sign test was used to determine whether participants' scores were significantly > 2 (‘*fair’*) on the 5-point scale.

## Results

### Sample characteristics

Participants in the TelePain-EVP (*n* = 221) ranged from 24 to 81 years of age. These veterans were typically male (65.16%), black or African American (76.47%), married or partnered (41.63%), attended at-least some college or vocational school (67.87%), and reported low back as their primary pain location (29.41%). Of the total 30 TelePain-EVP sessions, the median attendance for participants was 27 (*iqr* = 12) sessions. Participant demographic information is presented in Table 3.

**Table 3:** Demographic information for TelePain-Empower Veterans Program (EVP) participants (*n* = 221)

Characteristic	
Age (years), *m* ± *sd*	54.40 ± 10.80
EVP Dose, *mdn(iqr)*	27.00 (12.00)
Satisfaction with EVP*, *mdn(iqr)*	4.00 (2.00)
Gender, *n* (%)	
Female	77 (34.84%) 144
Male	(65.16%)
Race, *n* (%)	
Black/African American	169 (76.47%)
White/Caucasian	41 (18.55%)
Other	6 (2.71%)
Missing/Decline to Respond	5 (2.26%)
Marital, *n* (%)	
Divorced, Separated, or Widowed	48 (21.72%)
Married or Partnered	92 (41.63%)
Never Married	23 (10.41%)
Missing/Decline to Respond	58 (26.24%)
Employment, *n* (%)	
Disability	59 (26.70%)
Employed, < 40 Hours/week	14 (6.33%)
Employed, ≥ 40 Hours/week	23 (10.41%)
Unemployed, Looking for Work	11 (4.98%)
Unemployed, Not Looking for Work	15 (6.79%)
Retired	39 (17.65%)
Student	2 (0.90%)
Missing/Decline to Respond	58 (26.24%)
Education, *n* (%)	
High School	18 (8.14%)
Some College/Vocational School	45 (20.36%)
Associate degree	36 (16.29%)
Bachelor’s Degree	43 (19.46%)
Graduate Degree	26 (11.76%)
Missing/Decline to Respond	53 (23.98%)
Combat Veteran, *n* (%)	
No	96 (43.44%)
Yes	64 (28.96%)
Missing/Decline to Respond	61 (27.60%)
Primary Pain Location, *n* (%)	
Abdomen	3 (1.36%)
Arm/Hand/Fingers	11 (4.98%)
Face/Head	14 (6.33%)
Foot/Knee/Legs/Toes	34 (15.38%)
Low Back	65 (29.41%)
Mid Back	10 (4.52%)
Neck	17 (7.69%)
Shoulder	5 (2.26%)
Multi/Total Body	7 (3.17%)
Missing/Decline to Respond	55 (24.89%)

*iqr* Interquartile range, *m* Mean, *mdn* Median, *sd* Standard deviation

### Primary ePRO models

Data from time 4 (52 weeks) was excluded secondary to low response rate (*n* = 25; 3.79%). The LMMs examining primary and secondary outcomes focused on the fixed main effects of time and dose as well as their interaction. None of these models produced a significant time x dose interaction effect after accounting for the adjusted significance threshold (*p* < .01). These interaction terms were excluded from final models to preserve degrees of freedom and protect against of potential multicollinearity inflating standard errors.

#### Main effects of time for primary outcomes

**Fig. 2:**
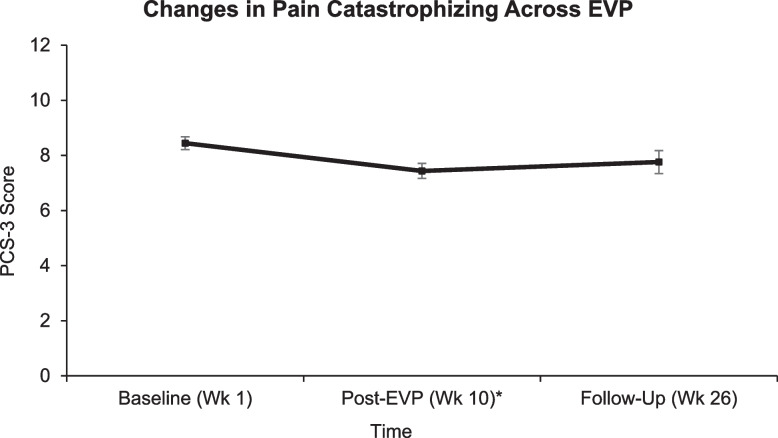
Changes in pain catastrophizing across TelePain-EVP. *Note.* Least squares means and standard errors for pain catastrophizing with lower scores indicating improvement. EVP = Empower Veterans Program; PCS-3 = Pain Catastrophizing scale, 3-item version; Wk = Week. *p < .01

Primary pain-related outcomes were pain intensity, interference, catastrophizing, and kinesiophobia. There was a significant medium effect size decrease in pain catastrophizing from baseline to post-Tele-Pain-EVP (*SMD* = -.345, *p* < .001). However, this improvement reducted to a small effect size and was non-significant from baseline to week 26 (*SMD* = -.204, *p* = .116), see Fig. 2.

A small effect size improvement was observed for pain interference from baseline to post-treatment (SMD = -.256, *p* = .05), but this improvement did not exceed the adjusted significance threshold. Pain interference at week 26 did not significantly differ from baseline with a modest effect size (*SMD* = -.102, *p* = .427). No significant changes for pain intensity or kinesiophobia were observed post-TelePain-EVP (*SMD* = -.059, *p* = .538) or at week 26 (*SMD* = -.127, *p* = .326). Results from the fixed main effects LMMs are presented in Table 4.

**Table 4:** Fixed effects estimates for patient-reported outcome measures

Outcome	Fixed Effects	*p*	95% *CI*		
	β ± *SE*		*LB*	*UB*	*SMD*
**Pain**					
* Intensity*					
Time 1^a^	-.132 ± .122	.281	-.372	.107	-.103
Time 2^b^	.109 ± 0.153	.477	-.191	.409	.092
EVP Dose	-.018 ± .010	.075	-.039	.002	
* Interference*					
Time 1	-.864 ± .439	.050	-1.724	-.004	-.256
Time 2	-.447 ± .561	.427	-1.546	.653	-.102
EVP Dose	-.034 ± .035	.335	-.101	.034	
* Catastrophizing*					
Time 1	-1.009 ± .275	< .001^*^	-1.548	-.470	-.345
Time 2	-.686 ± .434	.116	-1.536	.165	-.204
EVP Dose	-.036 ± .020	.083	-.076	.004	
* Kinesiophobia*					
Time 1	-.204 ± .332	.538	-.854	.446	-.059
Time 2	-.455 ± .462	.326	-1.360	.451	-.127
EVP Dose	.008 ± .023	.734	-.037	.052	
**Physical**					
* Physical Function*					
Time 1	-.690 ± .256	.009^*^	-1.199	-.180	-.249
Time 2	-.262 ± .398	.511	-1.042	.518	-.086
EVP Dose	-.027 ± .024	.261	-.074	.020	
* Sleep Disturbance*					
Time 1	.103 ± .140	.464	-.172	.377	.070
Time 2	-.189 ± .140	.178	-.462	.085	-.171
EVP Dose	-.007 ± .007	.340	-.021	.007	
**Psychological**					
* Anxiety*					
Time 1	-.446 ± .167	.008^*^	-.773	-0.118	-.256
Time 2	-.273 ± .246	.269	-.756	0.210	-.143
EVP Dose	-.021 ± .013	.116	-.047	0.005	
* Depression*					
Time 1	-.534 ± .173	.002^*^	-.872	-0.196	-.296
Time 2	-.322 ± .235	.172	-.783	0.139	-.175
EVP Dose	-.013 ± .013	.313	-.038	0.012	
* Motivation*					
Time 1	-.465 ± .308	.133	-1.069	0.139	-.146
Time 2	.019 ± .343	.752	-.564	0.782	.042
EVP Dose	-.013 ± .017	.451	-.021	0.046	
* Self-Efficacy*					
Time 1	.138 ± .327	.673	-.503	0.779	.041
Time 2	.019 ± .399	.962	-.762	0.801	.007
EVP Dose	.048 ± .019	.103	.012	0.085	
**Acceptance**					
* Activity Engagement*					
Time 1	1.977 ± .524	< .001^*^	.950	3.004	.364
Time 2	1.915 ± .658	< .001^*^	.626	3.204	.376
EVP Dose	-.034 ± .039	.378	-.110	0.042	
* Pain Willingness*					
Time 1	-1.041 ± .749	.032	-1.985	-0.098	-.209
Time 2	-.400 ± .481	.413	-1.355	0.556	-.106
EVP Dose	-.061 ± .030	.047	-.120	-0.001	
**Social**					
* Social Isolation*					
Time 1	-.850 ± .438	.054	-1.707	0.008	-.183
Time 2	-.421 ± .558	.452	-1.515	0.674	-.096
EVP Dose	-.052 ± .034	.134	-.119	0.016	

Statistically significant (*p* < .01)

*CI* Confidence Interval, *LB* Lower Bound, *UB* Upper Bound, *SE* Standard Error, *SMD* Standardized Mean Difference

^a^Change from baseline (Week 1) to post-TelePain-EVP (Week 10)

^b^Change from baseline (Week 1) to Follow-Up (Week 26)

#### Main effects of dose on primary outcomes

Two non-significant trends were observed for EVP dose across primary outcomes. Specifically, higher EVP dose (hours attended) corresponded with lower pain intensity (*p* = .075) and pain catastrophizing (*p* = .083) score. This was not the case for pain interference (*p* = .335) or kinesiophobia (*p* = .734). Overall, dose failed to achieve statistical significance for primary measures.

### Secondary ePRO models

Secondary outcomes covered multiple domains including physical, psychological, acceptance, and social. Again, none of the models produced significant time x dose interaction effects that exceeded the adjusted significance threshold. These interaction terms were excluded from final models.

#### Main effects of time for secondary outcomes

##### Physical

Participants' physical functioning scores decreased from baseline to post-TelePain-EVP indicating a small effect (*SMD* = -.249, *p* = .009). However, physical functioning scores were similar to baseline levels by week 26 (*SMD* = -.086, *p* = .511). Sleep disturbance scores did not change from baseline to post-treatment (*SMD* = .070, *p* = .464) or at week 26 follow-up (*SMD* = -.171, *p* = .178).

##### Acceptance

**Fig. 3:**
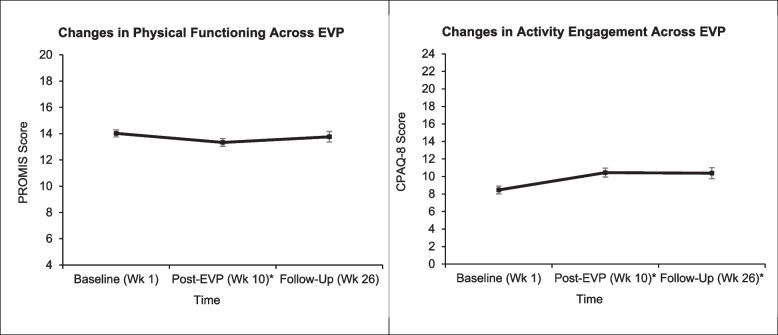
Changes in physical functioning (left) and activities engagement (right) across TelePain-EVP. *Note.* Least squares means and standard errors for physical functioning (left) and activities engagaement (right) with higher scores indicating improvement. EVP = Empower Veterans Program; PROMIS = Patient Reported Outcomes Measurement Information System; Wk = Week. *p < .01

Contrary to physical functioning, engagement in life activities, despite experiencing pain, increased from improved from baseline to post-TelePain-EVP (SMD = .364, *p* < .001). Furthermore, this medium effect size improvement remained significant at week 26 (*SMD* = .376, *p* < .001). Pre-post improvements in participants' willingness to experience pain without attempts to control it were also observed (*SMD* = -.209, *p* = .032), indicating a small effect. However, this effect did not exceed the adjusted significance threshold. No differences were observed from baseline to week 26 with an observed modest effect size (*SMD* = -.106, *p* = .416). See Fig. 3.

##### Psychological

Veterans’ anxiety (*SMD* = -.256, *p* = .008) and depression (*SMD* = -.296, *p* = .002) severity both decreased from baseline to post-treatment indicating small effects. However, anxiety (*SMD* = -.143, *p* = .269) and depression (*SMD* = -.175, *p* = .172) scores were not significantly different from baseline at week 26 follow-up. No improvements in motivation (all *SMD* = -.146, *p* = .133) or self-efficacy to manage pain (*SMD* = -.209, all *p* = .672) were observed post-treatment. These non-significant effects remained for motivation (*SMD* = .042, *p* = .753) and self-efficacy (*SMD* = .007, *p* = .962) were also observed at week 26. 

##### Social

**Fig. 4:**
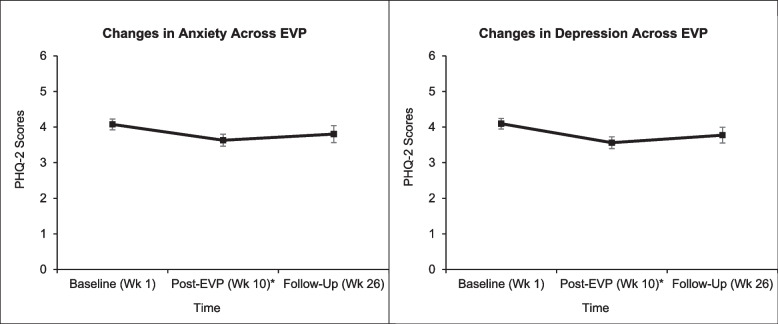
Changes in physical functioning (left) and activities engagement (right) across TelePain-EVP. *Note.* Least squares means and standard errors for anxiety (left) and depression (right) with higher scores indicating improvement. EVP = Empower Veterans Program; PROMIS = Patient Reported Outcomes Measurement Information System; Wk = Week. *p < .01

Veterans’ social isolation scores decreased from baseline to post-EVP (*SMD* = -.0183, *p* = .054), but this improvement was a modest effect and failed to achieve statistical significance. This trend did not remain at week 26 (*SMD* = -.096, *p* = .452). See Fig. 4.

#### Main effects of dose on primary outcomes

The effects of EVP dosing on secondary outcomes were also limited. Greater pain willingness (acceptance domain) was associated with higher EVP dose (*p* = .047), but this effect did not exceed the adjusted significance threshold. No significant dosing effects were observed for the physical, psychological, or social domains (all *p* ≥ .103). See Table 4.

#### Satisfaction with TelePain-EVP

Veterans’ satisfaction with TelePain-EVP scores post-treatment was skewed with many scores toward higher end of the 5-point scale. Participants had a median satisfaction with TelePain-EVP of 4.00 (*iqr* = 2.00). Results from a Wilcoxon Sign-Rank Test indicates that participants' satisfaction scores were significantly higher than the *fair* satisfaction rating (2.00) in the scale (*p* < .001). This finding indicates positive satisfaction with TelePain-EVP.

## Discussion

The VA response to the opioid epidemic includes the need to examine effective non-pharmacological programs for veterans with chronic pain. Emerging telehealth initiatives such as TelePain-EVP aim to increase access to chronic pain management programs for this high-risk population. Evaluation data indicate the TelePain-EVP has positive outcomes including small effects sizes for certain pain (catastrophizing), psychological (anxiety, depression), and a medium effect size acceptance (activities engagement) outcomes for veterans with chronic pain. Only the medium effect size improvement in veteran engagement in life activities despite pain (acceptance) persisted at week 26 follow-up. Interestingly, aside trends for a few variables (pain intensity, catastrophizing, acceptance – pain willingness), which failed to reach statistical significance, there were no TelePain-EVP dose-related improvements. However, veterans reported high satisfaction with TelePain-EVP post-treatment.

Primary pain-related outcomes for TelePain-EVP have been examined in EVP prior to its implementation to a telehealth program [36, 40]. In the current evaluation, pain catastrophizing was lower post-treatment compared to baseline with an observed medium effects size which was consistent with these previous evaluations. The lack of pre-post improvements in pain intensity [36, 40] and interference [36] were contradictory to previous evaluations which produced small and medium effects, respectively. However, neither of these previous studies examined follow-up time points so we were unable to make any comparisons beyond post-treatment. Specifically, the limited clinical improvements observed at week 26 follow-up, could have been impacted by the change in mode of delivery. These findings warrant consideration in future efforts to evaluate EVP. Of note, prior to COVID-19, EVP offered post-intervention skills groups to participants to help maintain participant outcomes. These skills groups are currently being adapted to the TelePain-EVP model.

Secondary outcomes compared to a previous pre-post evaluation of in-person EVP [36] are consistent; pre-post physical functioning decreased during TelePain-EVP similar to in-person EVP with small effects observed in each study. However, the fact that this negative finding did not persist at week 26 follow-up suggests that impact may only be temporary. Also, consistent with previous work, psychological outcomes, anxiety, and depression showed pre-post improvements. However, these small effect sizes were not significant at week 26. For in-person EVP the effect size for pre-post improvements in depression was medium, but this may be attributed to differences in outcome surveys between evaluations (PHQ-2 vs. PHQ-9) increasing variability of participants' responses [36]. Unlike previous work, sleep disturbance did not improve at any time point compared to the small effect size observed during in-person EVP [36]. Motivation and self-efficacy also did not improve at any time point and these metrics were unique to the current evaluation. Finally, activities engagement significantly improved (medium effect) from baseline to post-treatment which was consistent with previous work, though in-person EVP produced a large pre-post effect for this outcome [36]. This medium effect size for activities engagement observed during TelePain-EVP demonstrated robustness as evidenced by its maintained significance at week 26. The non-significant trend for improved pain willingness demonstrated a small effect size and is in the same direction of previous work (medium effect size improvement) supporting improved pain willingness pre-post EVP [36]. However this trend was not present 26 weeks from baseline. A non-significant trend indicated that social isolation reduced from pre to post-TelePain-EVP with a modest effect size, but not at follow-up. The latter effect is supported by a previous evaluation conducted by this team, indicating that social HRQoL improved from pre to post-EVP with a small effect size being observed [36]. These findings present the importance of the delivery mode preferences. While many participants may prefer remote access to EVP, noted drawbacks may include lower survey response rates, smaller pre-post effects size improvements, and others may prefer in-person contact. It is becoming apparent in the COVID-19 climate, though remote access to care is critical, people often crave interpersonal connection. Furthermore, increased prevalence of psychological disorders (e.g., generalized anxiety) during COVID-19 may have introduced increased complications for veterans served by TelePain-EVP that were less prevalent in previous evaluations [62].

The limited dose trends observed for TelePain-EVP were mixed and somewhat counterintuitive across many of the measures under study. This is consistent with a previous EVP evaluation which examined dose as a less granular, binary outcome (graduation) [36]. While not significant, TelePain-EVP dose was associated with lower pain intensity and catastrophizing. This may be due to veterans that have less severe pain intensity and negative cognitions about whether their severe pain could be managed were more likely to attend TelePain-EVP, an acceptance-driven intervention. Conversely, dose of the intervention was associated with higher pain willingness. However, for each of these outcomes, attendance failed to produce significant improvements. A likely reason for the limited dose-related outcomes was that dose was skewed in our evaluation sample indicating veterans that completed ePRO surveys were more likely to attend sessions. Perhaps outcomes may be better understood if our sample size and response rates were higher among veterans that did not complete these surveys. Such results may give a better indication as to the true impact of TelePain-EVP dose on outcomes. As such, outcomes related to measures are not mutually exclusive with outcomes. Collectively, past and current EVP evaluations suggest this program provides a viable pain management option with impacts on pain management outcomes; and telehealth is a feasible mode for EVP delivery.

When interpreting these data, the following limitations should be considered. First, this was a pragmatic quality improvement evaluation thus, the lack of randomized and/or blind controlled methods leaves the data vulnerable to self-selection/referral bias and confounded influence. Though findings indicate a significant improvement for TelePain-EVP, absence of a control group limit generalizability. It is notable that veterans referred to TelePain EVP typically have not responded well to previous pain care and present with additional complex issues (e.g., depression, opioid use, possible suicidality secondary to pain) [43], and identifying a control group for this unique population is very difficult and potentially unethical (e.g., suicidality). In attempt to ethically evaluate the impacts of the EVP without the benefit of a control group, it is notable that patients with chronic, sometimes comorbid needs, present a complex dynamic that can confound intervention effects. Yet, patients with chronic complex needs represent a population with the greatest need, and potential for much needed benefit, from whole health-oriented modalities which take a holistic approach to improving outcomes. Paradoxically, the complex nature of their condition and symptoms, may limit the change in outcomes in spite of high program satisfaction rates.

The single site, representing a VA in the southeast, may also limit generalizability. Alternatively, the representation of minority veterans, adds meaningful data to the literature for a historically under-represented demographic. Next, the use of self-reported data, though it is the accepted measure for pain outcomes, it is nonetheless subjective in nature. Finally, the low response rate at week 26 follow-up resulted limited statistical power for detecting significant effects (Type II error rate). The lower response rate at week 26 may also result in inflated effects sizes and lower reproducibility of results [63]. Future studies should integrate objective outcome measures such as wearable devices to triangulate with subjective data sources [64]. Future research should focus on identifying patient profiles who will most/least benefit from the TelePain-EVP approach to pain management, compared to other TelePain management programs [33, 34] offered within the VA system. Continued implementation and evaluation efforts are warranted to support the spread and sustainability of this TelePain management program.

## Conclusions

As the VA continues efforts to deliver accessible nonpharmacological pain management options, these evaluation data indicate that TelePain-EVP has positive outcomes for certain pain (catastrophizing), psychological (anxiety, depression), and acceptance (activities engagement) for veterans with chronic pain. More TelePain related studies are needed along with comparative and cost effectiveness methods to determine patient benefits and the economic value gained in the effort to integrate nonpharmacological treatment options into healthcare delivery.

### Supplementary Information

**Supplementary Material 1. **

## Data Availability

The datasets developed and/or analyzed during the current project available from the corresponding author on reasonable request.

## Abbreviations

ACT: Acceptance and Commitment Therapy
CI: Confidence Interval
CPAQ: Chronic Pain Acceptance Questionnaire
ePRO: Electronic Patient-Reported Outcomes
EVP: Empower Veterans Program
HRQoL: Health-Related Quality of Life
IQR: Interquartile Range
LMMs: Linear-Mixed Models
MDN: Median
MILES: Motion is Lotion Exercises
MM: Mindful Movement
PCS: Pain Catastrophizing Scale
PHQ-9: Patient Health Questionnaire-9
PMOP: Offices of Pain Management, Opioid Safety, and PDMP
PROMIS: Patient-Reported Outcome Measurement Information System
VA: Department of Veterans Affairs
WH: Whole Health
