# Supplementary material for: Benchmark findings from a veteran electronic patient-reported outcomes evaluation from a chronic pain management telehealth program
Source: BMC Health Serv Res. 2024 Mar 28;24:388. doi: 10.1186/s12913-024-10816-4 (PMC10976697; doi:10.1186/s12913-024-10816-4)
Supplement: Supplementary file 1 — Supplementary Material 1. [file 12913_2024_10816_MOESM1_ESM.docx]

Supplemental Digital Content 1. Raw means, standard deviations, and observations for primary and secondary patient-reported outcomes across TelePain-EVP time points.

| Scale | Statistic | Time | | | |
| --- | --- | --- | --- | --- | --- |
|  |  | Baseline (W1) | Post (W10) | Follow-Up (W26) | Follow-Up (W52) |
| **Pain** |  |  |  |  |  |
| Intensity | *m* ± *sd* | 7.3 ± 1.5 | 7.1 ± 1.5 | 7.5 ± 1.4 | 7.2 ± 2.1 |
|  | *n* | 136 | 110 | 60 | 23 |
| Interference | *m* ± *sd* | 12.8 ± 5.1 | 11.6 ± 5.1 | 12.4 ± 4.9 | 12.8 ± 4.7 |
|  | *n* | 129 | 112 | 61 | 23 |
| Catastrophizing | *m* ± *sd* | 8.5 ± 2.9 | 7.1 ± 3.2 | 7.8 ± 3.5 | 8.2 ± 2.1 |
|  | *n* | 132 | 113 | 60 | 21 |
| Kinesiophobia | *m* ± *sd* | 11.7 ± 3.3 | 11.7 ± 3.2 | 11.4 ± 3.3 | 11.2 ± 3.9 |
|  | *n* | 127 | 110 | 60 | 21 |
| **Physical** |  |  |  |  |  |
| Physical Functioning | *m* ± *sd* | 13.9 ± 3.3 | 13.1 ± 3.8 | 14.0 ± 3.7 | 14.0 ± 3.6 |
|  | *n* | 135 | 113 | 59 | 22 |
| Sleep Disturbance | *m* ± *sd* | 8.0 ± 1.0 | 8.1 ± 1.3 | 7.8 ± 1.0 | 8.1 ± 1.0 |
|  | *n* | 130 | 109 | 62 | 22 |
| **Psychological** |  |  |  |  |  |
| Anxiety | *m* ± *sd* | 4.1 ± 1.8 | 3.4 ± 2.0 | 3.8 ± 2.2 | 3.7 ± 1.9 |
|  | *n* | 129 | 109 | 60 | 20 |
| Depression | *m* ± *sd* | 4.1 ± 1.8 | 3.4 ± 2.0 | 3.9 ± 2.1 | 3.9 ± 2.1 |
|  | *n* | 131 | 109 | 61 | 21 |
| Motivation | *m* ± *sd* | 8.1 ± 2.6 | 7.7 ± 2.5 | 8.2 ± 2.2 | 7.9 ± 2.6 |
|  | *n* | 127 | 107 | 57 | 21 |
| Self-Efficacy | *m* ± *sd* | 4.9 ± 3.0 | 5.3 ± 2.5 | 5.1 ± 2.7 | 5.3 ± 2.4 |
|  | *n* | 126 | 107 | 57 | 21 |
| **Social** |  |  |  |  |  |
| Social Functioning | *m* ± *sd* | 12.8 ± 5.1 | 11.6 ± 5.1 | 12.4 ± 4.9 | 12.8 ± 4.7 |
|  | *n* | 129 | 112 | 61 | 23 |
| **Acceptance** |  |  |  |  |  |
| Activity Engagement | *m* ± *sd* | 8.5 ± 5.3 | 10.7 ± 5.9 | 10.0 ± 5.6 | 10.5 ± 4.8 |
|  | *n* | 130 | 107 | 60 | 21 |
| Pain Willingness | *m* ± *sd* | 18.1 ± 4.4 | 16.9 ± 4.4 | 17.8 ± 4.4 | 17.9 ± 4.9 |
|  | *n* | 130 | 107 | 60 | 21 |

*EVP = Empower Veterans Program; iqr* = Interquartile range; *m = Mean; mdn* = Median; *sd = Standard Deviation; W1 = Week 1; W10 = Week 10; W26 = Week 26; W52 = Week 52*
